# Proof of impact and pipeline planning: directions and challenges for social audit in the health sector

**DOI:** 10.1186/1472-6963-11-S2-S16

**Published:** 2011-12-21

**Authors:** Neil Andersson

**Affiliations:** 1 Centro de investigación de Enfermedades Tropicales (CIET), Universidad Autónoma de Guerrero, Calle Pino, El Roble, Acapulco, México

## Abstract

Social audits are typically observational studies, combining qualitative and quantitative uptake of evidence with consultative interpretation of results. This often falters on issues of causality because their cross-sectional design limits interpretation of time relations and separation out of other indirect associations.

Social audits drawing on methods of randomised controlled cluster trials (RCCT) allow more certainty about causality. Randomisation means that exposure occurs independently of all events that precede it – it converts potential confounders and other covariates into random differences. In 2008, CIET social audits introduced randomisation of the knowledge translation component with subsequent measurement of impact in the changes introduced. This “proof of impact” generates an additional layer of evidence in a cost-effective way, providing implementation-ready solutions for planners.

Pipeline planning is a social audit that incorporates stepped wedge RCCTs. From a listing of districts/communities as a sampling frame, individual entities (communities, towns, districts) are randomly assigned to waves of intervention. Measurement of the impact takes advantage of the delay occasioned by the reality that there are insufficient resources to implement everywhere at the same time. The impact in the first wave contrasts with the second wave, which in turn contrasts with a third wave, and so on until all have received the intervention. Provided care is taken to achieve reasonable balance in the random allocation of communities, towns or districts to the waves, the resulting analysis can be straightforward.

Where there is sufficient management interest in and commitment to evidence, pipeline planning can be integrated in the roll-out of programmes where real time information can improve the pipeline. Not all interventions can be randomly allocated, however, and random differences can still distort measurement. Other issues include contamination of the subsequent waves, ambiguity of indicators, “participant effects” that result from lack of blinding and lack of placebos, ethics and, not least important, the skills to do pipeline planning correctly.

## Introduction

CIET social audit methods originated in the mid-1980s in Central America [1-3] as an attempt to generate reliable evidence on the coverage of key interventions and their presumptive outcomes. This first generation of social audit focussed on simple indicators and stakeholder discussions about what these might mean and what could be done about them [4]. The aim was not to prove causality, but to begin discussions with communities and planners about what worked and how.

A second generation of social audits strengthened methods of feedback of evidence to stakeholders in communities and health services, and collated a *second order of evidence*: what communities and service workers felt could be done about the problems identified in the household surveys. Just as we aggregated, for example, low vaccination rates and costs of measles [5] across an epidemiological sample, we could aggregate community-led solutions to these problems.

These first two generations of social audits relied on observational studies that combined qualitative and quantitative uptake of evidence with consultative interpretation of results. Because of this design, subsequent discussions often stumbled over issues of causality.

In 2008, as part of the five year Nigeria Evidence-based Health System Initiative (NEHSI) in Bauchi and Cross River states [http://www.idrc.ca/EN/Programs/Global_Health_Policy/Governance_for_Equity_in_Health_Systems/Pages/ProjectDetails.aspx?ProjectNumber=104613], social audits introduced randomisation of the knowledge translation component, with subsequent measurement of impact of the changes introduced. The state governments nominated maternal outcomes as their first health priority for study. In each state, a random sample of 60 sites provided state level representation for a baseline survey of maternal morbidity. In addition, 10 randomly selected sites in each of three randomly selected focus local government authorities (LGA) in each state, provided increased sensitivity of local analysis. Preliminary analysis involved dozens of state and LGA level health managers, who analysed and discussed results in a training setting. Field teams took the emerging evidence into gender stratified discussions in each of the 180 sites. Facilitators asked questions and used standardized prompts to elicit responses.

The most consistent and prominent of 28 candidate risk factors for non-fatal maternal morbidity was intimate partner violence (IPV) during pregnancy (ORa 2.15, 95%CIca 1.43-3.24 in Bauchi and ORa 1.5, 95%CI 1.20-2.03 in Cross River). Other spouse-related factors in the multivariate model included not discussing pregnancy primarily with the spouse and, independently, IPV in the last year. The social audit concluded that the violence women experience throughout their lives – genital mutilation, domestic violence, and the effects of steep power gradients – is accentuated through pregnancy and childbirth, when women are most vulnerable. IPV especially in pregnancy, women's fear of husbands or partners and not discussing pregnancy are all within men's capacity to change. Few women had a say in where they would deliver their children.

These preliminary results fed into gender-stratified focus group discussions in each site. Sharing the results with participants, facilitators asked questions and used standardized prompts and monitors recorded the separate male and female discussions about work during pregnancy, safe pregnancy and safe birth, IPV and female genital mutilation (FGM).

The preliminary analysis and focus group discussions focussed on solutions that increase male responsibility around pregnancy and childbirth. First, a video drama (separate for each state) showed a woman with pregnancy problems mistreated by her husband; this supported structured discussions within each site, to arrive at a series of local solutions. Second, local health workers received a scorecard of local indicators related to maternal morbidity, and viewed the video drama. They also received training in primary prevention strategies focussed on men. Third, all pregnant women in the sample sites received several visits from health workers and traditional birth attendants. They asked about current health, safety and access to care of each pregnant woman. A separate interview with the husband or partner asked about potential ways to reduce heavy work in pregnancy, the issue of violence in pregnancy, and how to encourage discussion of pregnancy between husband and wife.

In the sample based social audit, we intended these three strategies to generate community-led solutions that could feed in to LGA plans and, with the roll-up of the sample, state authorities. As it turned out, the process of generating the intervention itself had the potential to decrease maternal morbidity in the six randomly selected LGAs. This “proof of impact” is a *third order of evidence* produced in a cost-effective way, providing implementation-ready solutions for planners at state level.

### Quantitative evidence and emergence of pragmatic randomised controlled trials

In the 1990s, the New Public Administration approach in the USA attempted to make it normal to provide “definite and quantitative evidence” [6]. With the increasing demand for high quality evidence, methods of social audit [7] have evolved beyond public opinion polls and observational studies, to include techniques like randomised controlled trials (RCTs). These trials (experiments), which involve using comparisons with people or places that do not receive the intervention in question (controls), rely on random allocation of the intervention to generate convincing evidence of impact or lack of impact.

The international evaluation industry has heard loud voices of those who say impact is measurable and provable [8-10] -- among other ways, using RCTs -- and those who are overcome by the many practical and theoretical difficulties of this proposition [11-13].

It has become a cliché to claim that controlled trials are the only design that can account for selection bias and demonstrate a causal relationship between intervention and outcomes [14-16]. Several issues affect application of RCT methods in the area of policy. There may be ethical issues of withholding interventions in control communities [17]. Biases can result from health workers and communities alike knowing what the interventions are [18] and there are difficulties of appropriate analysis [19]. Other problems include the often changing contexts of interventions, logistical and practical challenges, difficulties with monitoring service delivery, access to the intervention by the comparison group and changes in selection criteria and/or the intervention over time [20].

Elaboration of pragmatic RCTs over the last 10-15 years has addressed and clarified many of the issues associated with RCT-related methods in the realm of policy [21-25].

Much of the difference between explanatory and pragmatic trials can be summarised by the questions they try to answer. An explanatory RCT might ask, “Can this vaccine have a protective effect under ideal conditions?” Pragmatic RCTs typically apply interventions we *know* to have an impact, perhaps from prior explanatory trials, and might ask the next question, “How well does this vaccine actually work in real life?” [26].

The emergence of pragmatic trials has implications for evidence-based planning. First, randomising and comparison with a control group are the principal contributions of pragmatic RCTs, not double blinding or placebos [27,28]. Apart from the considerable advantage of experiments that they arrange the exposure to precede the outcome, the pivotal value of randomising in health policy and planning is that this converts covariants into random differences that can be assessed formally using standard statistical procedures [29-31]. Randomisation means that everything associated with the outcome prior to the exposure is independent of the exposure. This shifts the onerous burden of proof of observational studies, where the researcher is obliged to exclude potential confounders one by one as possible explanations of a presumed effect.

An important advantage of pragmatic trials is the ability to contemplate real life costs to health services and to the intended beneficiaries. They also allow consideration of unintended effects, positive and negative. In most pragmatic trials, the unit of randomisation, intervention and analysis is the cluster: the community or a segment of it. These randomised controlled *cluster* trials (RCCT) have an interesting application in contemporary social audit.

### Pipeline planning and social audit

Social audits have incorporated the elements of pragmatic RCCTs; pipeline planning refers to the real time use of evidence from this type of social audit to fine tune interventions in the programme pipeline. The idea behind pipeline planning is simple and compelling: in the framework of a pragmatic RCCT, implement an intervention in an initial wave of randomly selected places; then, after an appropriate interval, measure the impact of this in comparison with the second wave, before they start the intervention. In successive waves of intervention, each is contrasted with a previous wave. Each new wave and measurement generates high quality evidence on impact and intermediate outcomes (such as risk behaviours). This information can feed into adjustments of the next wave of the intervention – the pipeline -- fine tuning it to optimise its impact.

As an example, a concern to deal with unofficial payments and staff attitudes in health services might begin with a national baseline survey that documents payments and views of the public about government services [5,32-35]. Informed by this community input, candidate interventions might be discussed with service workers and planners. These can be implemented in an initial wave of sites – perhaps the original sample sites – and then rolled out through successive waves, the baseline of each wave on commencement serving as the “control” for the last intervention wave. A similar approach can be relevant to roll out of interventions across a wide range of health issues – HIV prevention, diabetes or hypertension care.

In parallel with the rollout of the intervention, a dialogue with research users (in our social audits, planners, service workers and communities) should increase engagement on emerging evidence of the impact of the candidate intervention; cultural and contextual aspects to prevention, especially local (if forgotten) resilience behaviours in the various cultural groups; neglected aspects of current prevention efforts, for example choice disability (when people are unable to implement protective choices).

The stepped-wedge RCCT is a pragmatic trial design that compares an initial wave of randomly selected individuals or clusters with successive waves that join the intervention at intervals, until all have been included. Random allocation of similar sets to each wave has the same effect as randomisation in a simple randomised controlled trial: exposure occurs independent of all the events that precede it. The key assumption is that each wave selected to receive the intervention in the future is similar to the initial intervention, and therefore comparable in terms of the outcome of interest.

### Technical aspects: pipeline planning of the reduction of maternal morbidity in Nigeria

The same three components of community engagement on maternal morbidity in Nigeria will be rolled out as a stepped-wedge randomised controlled trial across the remaining 17 LGAs in Bauchi state; the intervention was piloted in three randomly selected LGAs. This will be conducted in two waves, two years apart, with eight LGAs randomly assigned to each wave. Implementation detail of this helps to illustrate the mechanisms and logistics of pipeline planning.

A brief baseline household census will contact every household in the LGA, to identify women who have been pregnant in the last two years and women who are currently pregnant. The interviewer will ask those who have been pregnant in the last year to provide information about risk factors and maternal morbidity, using a shortened version of the social audit questionnaire. In parallel, all clinic and health facility records will be collated and reviewed to establish the maternal mortality in the LGA in the previous two years. Supplementary sources of information about maternal mortality will include funeral parlours and religious leaders who conduct burials. In each LGA, the programme will involve several steps geared to increase acceptance of the responsibility of men in maternal morbidity and mortality.

Evidence from the social audit will be shared with all residents of the first randomly selected LGAs (eight per state) through the three channels developed during the Socialising Evidence for Participatory Action (SEPA) phase of the social audit:

1. the video drama will be shown in all communities in the LGA, and used on local television and in schools to generate discussions about men and pregnancy;

2. health workers across the entire LGA will receive orientation on primary prevention of maternal morbidity, in addition to revision of danger signs in pregnancy, and their management;

3. every pregnant woman and her husband or partner, identified during the baseline survey, will receive several visits in the course of the pregnancy; these visits will extend to other women who become pregnant, identified through networking among pregnant women.

At the end of two years, a repeat census will contact every household in the first wave LGAs and all households in the second wave LGAs. Again, this will identify women who have been pregnant in the last two years, and those currently pregnant. As in the baseline, collation of health facility records, funeral homes and religious leaders will attempt to document maternal mortality. This repeat census will provide the baseline for the second wave will allow assessment of the impact of the intervention on maternal morbidity and mortality in the first wave.

After the baseline census in the first wave of LGAs, analysis of the difference between these and the six randomly selected pilot LGAs will allow for fine tuning of the intervention prior to its rollout. Again when the first wave and second wave are compared two years later, the analysis will inform fine tuning of the intervention, adding new components or modifying existing components. At this point it will also be possible to assess the first six randomly selected LGAs to see what happens two years after their intervention ended.

#### Sample size computations

In pipeline planning, sample size computations are illustrative of the level of power available rather than the part of the rationale for going ahead with the trial. In Bauchi state, there are around 800,000 households. The average LGA (40,000 households) should see around 10,000 pregnancies and 800 maternal deaths each year. Treating maternal mortality as a continuous variable (cluster size 10,000, SD 0.35, ICC 0.15, 0.8 power, 0.05 significance) we anticipate eight clusters per wave could detect a 20% reduction in maternal mortality. Because maternal morbidity is more common, affecting nearly one half of the women, this design would be adequate to pick up a 10% reduction in maternal morbidity (cluster size 10,000, SD 0.18, ICC 0.15, 0.8 power, 0.05 significance).

#### Logistics

The intervention is labour intensive, requiring the hiring and training of around 300 people in Bauchi state. In each wave, each household will receive one visit each year to identify pregnant women and outcomes in the previous two years. Each pregnant woman will receive four monthly visits each pregnancy. This comes to some 80,000 household visits each year per LGA – 10 visits per day, 20 working days per month, for each of 33 traditional birth attendants, community health extension workers and junior community health extension workers. Each wave would imply employment of 260-270 of these health workers across the eight LGAs.

#### Analysis

In a spirit of social audit rather than primary causality research, we are interested here in the whole package working together. The concern *is* causality, but in the sense of the impact of the intervention in real life, rather than the efficacy of a specific intervention under ideal conditions.

A 2006 systematic review of 12 stepped wedge controlled trials found no two trials used the same analysis approach [36]. Cluster randomised trials have two features that distinguish them from individual randomised trials. First, there can be positive intra-cluster correlation between individuals’ target behaviour outcomes within the same group. This can be due to the differences in characteristics between clusters or there may be interaction between individuals within the same cluster, particularly if there is a shared response to an intervention experienced by the whole cluster. Second, given that the intervention is delivered at the cluster level, the most cost-efficient design is to randomise a relatively small number of clusters to each intervention, and to have a moderate or large number of participants per cluster. This means we tend to overestimate the significance of differences between intervention and control waves.

Analysis in pipeline planning must acknowledge this. In trails with a single measure of outcome, analysis is straightforward. Among the commonly used analytic methods, GLMM works well but requires assumptions about the data distribution. GEE does not require these assumptions but can be too liberal, as it does not address inter-cluster variation. Feng and colleagues [37] recommend permutation-based inference which avoids distribution assumptions but it is not easy to explain to general users and it is not for planners with a general level of competence in statistics. For the social audit cluster sample, we have adapted the venerated and easy to explain Mantel Haenszel procedure, allowing for heterogeneity of effects across clusters with a robust odds ratio estimate [38]. This produces similar results to GLMM but without the assumptions or computational opacity. The Hussey and Hughes [39] approach to stepped wedge designs deals with changes over time in outcome variables and accounting for repeated measures on the same individuals over the duration of the trial.

### Problems and challenges

Exchanges between protagonists of RCTs and others who question their value and feasibility, have identified several issues that merit consideration.

*1. Controlled trials are not always enough*, *and not always necessary to prove causality*. The fact that an RCT demonstrates a positive effect of an intervention does not guarantee adoption of an intervention. Not all interventions can be randomised. For interventions that can be randomised, pipeline planning should not be about *discovery* of causality. We should not start pipeline planning without knowledge that the intervention should have a positive effect. Pipeline planning is rather about the amount of benefit from an intervention, its costs and side effects in practice, in the spirit of social audit. In this setting, randomisation is primarily about side-lining other explanations for an association between coverage and a change in health status, to focus on the number of cases the intervention saves in real life.

In the many cases where it is not possible to implement a new intervention or to reinforce existing interventions everywhere at the same time, randomisation can also be an equity issue. Assuming the intervention should eventually reach the whole country or region, random selection of groups, clusters or communities to waves of intervention gives everyone the same chance of early benefit (see equity issues).

Randomised trials bring problems of their own, however, and these must be more than balanced by the advantages before going ahead. Problems include contamination, ambiguity of indicators, “participant effects” that result from lack of blinding and lack of placebos, ethics and, importantly, the skills to do them properly. There is a positive side. Whereas analysis of linked cross sectional observational studies can be really complex, requiring considerable skill, analysis of a well designed and well implemented RCCT is relatively simple.

*2. Intervention development* is not an automatic or mechanical matter and communities do not always come up with the most effective solutions. Some might claim the government has to solve it for them. Others unrealistically expect a tertiary level hospital placed near them to solve their problems. Our approach to social audit does not view the epidemiologists as without knowledge. They know some things, and their job is to bring what they know to the table along with other expertise relevant to the discussion. The intervention most likely to succeed is likely to be supported in the literature or local knowledge. It will have buy-in from community, services and planners. Almost always, interventions require extensive piloting. In our current pragmatic RCCT on reduction of choice disability related to HIV in three southern African countries [40], piloting and packaging of just one of the three interventions took nearly two years. This development time can be reduced and formalised, as we did in Nigeria, using a proof-of-impact variant of social audit.

Timing of the impact requires consideration. In the Nigerian initiative, we allow two years for an effect to become measurable. As the women visited will be pregnant at the time of the visit, it is reasonable to conclude that reduction in maternal morbidity will be within the follow-up period. Many other interventions and outcomes, however, have a less predictable relation with time. Each step in a stepped wedge design may allow two years, where the impact takes three or four years to develop. This would lead planners wrongly to conclude the intervention is ineffective.

*3. Contamination* is not limited to controlled trials. The concern, in the Nigerian maternal morbidity example, is that communities in the second wave hear about the intervention and implement some of it. This decreases the contrast between them and the first wave. A similar dilution of measured effect happens if the intervention is not properly implemented. The cluster intervention design can help to reduce both effects. By using a unit of randomisation, intervention and analysis that is logistically and administratively coherent, spill over can be minimised. In the Nigerian case, the unit of randomisation and intervention is the local government authority (LGA), which is coterminous with several crucial health administrative functions. Although communities in neighbouring LGAs may come to hear of the programme, especially if it is successful, the effect of contamination is likely to be much less than if individual households were randomly allocated to intervention or control.

Perhaps the most famous and positive case of “contamination” is the Framingham study of ischaemic heart disease. This successfully moved the focus of attention “upstream” from specialist medical care to address the underlying lifestyle causes. A crucial lesson from the Framingham experience was in the way results translated rapidly and effectively into American and, some would say, global culture [41]. The resulting contamination, such that the Framingham cohort was not significantly different from the rest of the USA, is probably the biggest success of the initiative. It is impossible to avoid contamination in successful interventions, if what we're concerned with is improved health, nor should we be worried about it. What we should try to do, however, is document (i) mechanisms for generalising evidence, (ii) uptake at policy level and in the public discourse, (iii) impact in the control sites.

The efficiency of implementing in a cluster – covering 100 contiguous households rather than 100 randomly selected household scattered throughout the sample domain – increases the likelihood of proper implementation within a limited budget. In practice, it cannot be guaranteed that intervention and comparison groups are comparable in all respects, and additional steps may be necessary to verify comparability.

Stepped wedge designs, where an intervention rolls out to randomly selected waves of intervention groups or clusters, can minimise spill over, as the incentive to migrate to a different area just to benefit from the intervention is counterbalanced by the fact that this intervention is going to reach the entire study area within a known period of time – there is a pipeline. This design also increases understanding of the effect of time on the impact of an intervention [42].

In pipeline planning, interventions are almost never alone; they are in combination with existing interventions. In the case of maternal mortality, there are several federal and state government, NGO and private sector initiatives to reduce maternal mortality. For control units to “learn” from intervention units – LGAs in the Nigeria initiative – they first would have to identify the specific components and get involved with them. They might know there is a video drama. They might even view it. But if they have not discussed what they can do about it, in their community, with other members of their community, they are unlikely to implement the self-driven interventions that result from this in the intervention communities.

*4. Ambiguity of indicators* and difficulty in defining the measurement parameters are not solved by randomisation. Using a similarly flawed indicator in both intervention and control groups has at least the advantage that the error is constant. But this does not make it a better indicator; this task that must be tackled independent of study design. Our approach to this, during the design stage, is to open a consultation with the constituency – women, men, and health workers – about their understanding of the issue and their understanding of the terms. Rather than working backwards from “our” instrument, we have found it worthwhile to elicit their way of viewing the issue using approaches like fuzzy cognitive mapping (FCM) [43-45]. We have used FCM as an effective tool to review local knowledge and beliefs around a community health issue, contrasting the local belief system to that of Western science. This can go on to inform various stages of the research process, including the formulation of hypotheses, questionnaire development, and even data analysis [46]. With the categories and concepts identified in a participatory process, we first try to fit standard questions to each concept. If this does not do the job, we have to do the lengthy if interesting process of generating a new indicator.

*5. Blind*, *double blind and placebos:* The concern is that if people know they receive an intervention, as they certainly will do, they might change their behaviour *because* they received an intervention, rather than because of the specific content of the intervention. Blinding and placebos are not the primary concerns of pragmatic trials. People know that they are being vaccinated, or receiving a particular education regime in prenatal care, or attending a particular health centre – the challenge is to be sure it is not just this knowledge causing the effect. The easiest way to do this is to include the knowledge of exposure as part of the “package” getting tested. Pragmatic trials almost always address a complex intervention: If we make this vaccine available and promote its uptake through this or that approach, what is the preventive effect? In relation to male circumcision and HIV prevention, as another example, one would examine the impact of a programme that promotes circumcision; in an explanatory trial one would be more concerned with HIV occurrence in men who had accepted circumcision.

*Placebos* may be unnecessary and even unethical in the public health context, but there is nothing unethical about continuing or reinforcing current best practice in a control group. Although it is not realistic to do an educational intervention in one household and not in a neighbouring household, it is entirely feasible to do the intervention in one town, and not in another town. Efficacy trials have to be careful that placebos and blindness are not mistaken for trickery and deception. Pragmatic trials can bundle consent with the intervention, providing information about what the intervention is, its expected effects and possible side effects. The public come forward to take it up if they consent, and they do not if they do not consent.

*6. Research ethics* are a key issue in RCTs. Barahona draws attention to the stringent ethical codes associated with RCTs, and the need to get informed consent. He argues that this is difficult in group settings, especially if subjects are to remain blind to the exact intervention [47]. In pragmatic trials, consent is rarely the same issue as it is in an explanatory trial. Pragmatic trials involve interventions of proven efficacy, and an informational component can and should contain all the information needed for individual informed consent. Pragmatic trials are about real life choices. People need information about the pros and cons of any health service offer; then they can take it up or decline to do so. In a programme pipeline, where all communities will eventually benefit from the intervention, having a random order adds an equity component without the ethical dilemma of withholding a potentially beneficial intervention.

*7. Skills and infrastructure:* Pipeline planning requires redevelopment of the “info-structure” (information management and evidence synthesis behind planning) as an agile and responsive tool, a living and flexible component of planning able to combine qualitative and quantitative evidence on different levels of prevention. The Nigerian project provides this through the existing social audit programme, which has strong acceptance from the respective state governments of Bauchi and Cross River. In most settings, this will require considerable additional investment, especially outside of the national or state capitals. The need for skills goes beyond advanced epidemiology, though this is key. It includes appropriate use of evidence, a respect for evidence, and an increasing ability to distinguish between evidence and ideas. This content can be the focus of a series of brief executive workshops, involving parliamentarians and senior planners.

## Conclusion

RCTs, efficacy or pragmatic, are not the answer to every problem. Our next generation social audits draw on these experimental methods in proof-of-impact and pipeline planning not to discover causality, but to monitor impact in a way that is relatively free of the constraints of observational studies.

Introduction of high level research methods into programme roll out has multiple advantages, including improving the programme in the pipeline, but it requires a quantum shift in skills and sensibilities. In the short and middle term, third party NGOs and universities can provide some of this. Sustainability will depend on government buy in – which, in turn, depends on the system showing its value in terms of money saved and impact achieved.

Pipeline planning in its full application requires strong and sustained government interest. This is typically least present in situations that would most benefit from evidence-based planning. A step in the right direction, making use of external support and the limited scale this allows, is to implement a pragmatic RCCT in selected sites across a jurisdiction like a state or country. This allows for skill building to take it to scale and increases buy-in based on feasibility and positive results.

## Competing interests

The author declares he has no competing interests.
